# Clinical applications and research progress of totally implantable venous access ports: a literature review

**DOI:** 10.3389/fonc.2024.1519728

**Published:** 2025-01-16

**Authors:** Xiao-Min Huang, Xia Li, Jie Deng, Jiong Chen, Liang Qian

**Affiliations:** ^1^ Department of Anesthesiology, The Seventh Affiliated Hospital, Sun Yat-sen University, Shenzhen, China; ^2^ Centre for Respiratory and Critical Care Medicine, The University of Hong Kong-Shen Zhen Hospital, Shenzhen, China

**Keywords:** cancer, catheter, central venous access device, intravenous drug delivery system, subcutaneous injection, totally implantable venous access port

## Abstract

Totally implantable venous access port (TIVAP), a novel intravenous infusion system that is used for long-term intravenous treatment, has become increasingly popular among cancer patients undergoing chemotherapy and other patients requiring long-term intravenous infusions. This technology has been introduced into clinical practice in China, with successful results. Nevertheless, there are still certain problems; for instance, China has not set up a specialized regulatory agency to oversee research and set guidelines for the comprehensive life-cycle management of TIVAP. Additionally, there exists a disparity in standardized operations and complication management related to TIVAP, which has resulted in variable outcomes, complications, and patient satisfaction with TIVAP implantation across different medical units in China. Therefore, this article aims to provide a systematic overview of the clinical applications and maintenance of TIVAP, both domestically and internationally. Furthermore, this review investigated the latest strategies and associated research on TIVAP implantation and complication management, aiming to provide a basis for standardized surgical and maintenance procedures, protocols to minimize complications, and approaches for enhancing the overall quality of life for patients.

## 1 Introduction

In recent years, a subcutaneous device to deliver drugs, specially called the totally implantable venous access port (TIVAP), has been developed for long-term subcutaneous injection. This system enables the infusion of various fluids, including irritating therapeutic drugs, parenteral nutrition solutions, and other essential liquids (1). According to international guidelines, an infusion port is typically used for cancer patients who require long-term intravenous treatment or nutritional support and have poor peripheral vascular conditions (2, 3). Furthermore, considering the urgency of the situation and the availability of alternative methods for establishing central venous access, its contraindications must be carefully evaluated (4). Compared with other intravenous infusion systems, TIVAP has remarkable characteristics (5). It is composed of a combination of soft and easily foldable silicone or highly biocompatible polyurethane. The implantation methods for the infusion port involve a process of open vascular incision techniques and vascular percutaneous closed puncture techniques. Operators usually complete percutaneous closed perforation which could be implemented through one of three methods: ultrasound-guided technology, radiation-assisted technology, and anatomical marker positioning technology (6). To offer a profound understanding of the current progress in this field, we conducted a systematic review of the clinical applications and maintenance of TIVAP, both domestically and internationally, relying on publicly accessible literature.

## 2 Development of infusion technology

With the continuous development of medical technology, the concept of coexisting with tumors has gradually become widely accepted by the general public (7). Therefore, there is an urgent need to establish a method for long-term, reliable intravenous infusion access for cancer patients, which has become a key issue (8).

### 2.1 Application of superficial venous access catheters

In routine medical procedures, scalp needles and indwelling needles, specifically tailored for short-term and medium- to long-term infusions, have historically been the most widely adopted, economical, and minimally invasive infusion techniques (9). However, these procedures involve puncturing superficial veins in peripheral areas, and their use is often hindered by complications such as vascular occlusion and phlebitis, thereby limiting their wider application.

### 2.2 Application of central venous catheters

Due to the growing demand for long-term chemotherapy, parenteral nutrition support, repeated blood transfusions, and the application of irritating drugs in cancer patients, the implementation of central venous access catheters has gradually achieved broader application.

The first generation of long-term central venous access devices (CVADs) were designed by Broviac and Hickman in the 1970s (10, 11). Later, the development of central venous catheters (CVCs) accelerated, eventually leading to the successful clinical adoption of the first fully implantable intravenous infusion port in 1982 (12). Current studies have indicated that in the United States, more than 5 million people need to undergo central venous catheterization treatment each year (13).

According to their structure, CVCs are divided into two major categories: tunneled and non-tunneled. Within the tunneled category, there are two subdivisions: fully implanted and partially implanted. Currently, fully implanted tunneled TIVAP and non-tunneled peripherally inserted central catheters (PICC) and are the most commonly used options, respectively, as they provide long-term and stable intravenous infusion access (7).

### 2.3 Comparison of clinical efficacy between the totally implantable venous access port and peripherally inserted central catheters

Both TIVAP and PICC demonstrate stability in facilitating central venous access, although each method has its unique advantages and limitations. TIVAP is highly convenient for subcutaneous port implantation. However, the requirement for repeated punctures in a sterile environment may increase the patient’s pain; meanwhile, the use of a specific size of butterfly needle restricts the infusion rate (14).

In 2020, the team of Qi Fangmei et al. reported that the puncture success rate in the PICC group was 98.96%, and in the TIVAP group was 98.91%, with no statistically significant difference between the two. However, it was observed that the retention time in the TIVAP group was significantly longer than that in the PICC group, and the complication rate in the TIVAP treatment group was lower than that in the PICC group (15). Another report from Taxbro K et al. showed that in the PICC group, the risks for catheter-related thrombosis, infections, and various other complications were higher than those in the TIVAP group (16). It is reported that approximately the cost ($3,925.83) of TIVAP device implantation and removal was significantly higher than that of the PICC device ($957.14), because an operating room was needed for TIVAP. Conversely, the approximate costs for daily maintenance and complication management of the PICC device were greater than those of the TIVAP device. Considering a 6-month retention period, there was no significant difference in the overall treatment cost between PICC and TIVAP (16). Therefore, the choice of a CVAD should comprehensively consider the patient’s condition and economic ability.

## 3 Indications and contraindications of the totally implantable venous access port

Since a TIVAP is set up via the internal jugular vein or the subclavian vein with a tunneled catheter, it can be removed at any time when the treatment is over, serious complications arise, or the patient is unable to tolerate them (17).

### 3.1 Advantages of the totally implantable venous access port

The TIVAP has achieved greater acceptance among patients due to its implantable structure, as a patient receiving TIVAP can freely engage in normal activities without the worry of disrupting the device. Unlike some other CVADs, they don’t have to visit the hospital frequently for dressing changes or tube maintenance. Moreover, TIVAP has a lower occurrence rate of catheter-related thrombosis and infection when compared to other CVADs. These factors lead to less inconvenience and a better quality of life for the patient.

### 3.2 Indications for the totally implantable venous access port

Indications for clinical use of the TIVAP include (1): Long-term intermittent infusion of corrosive, irritating, or highly permeable drugs (2); The need for long-term parenteral nutrition support (3); Poor peripheral vascular conditions exist, yet long-term repeated blood drawing or medication injection is necessary (4); Long-term repeated infusion of blood products is demanded (5); The patient has a strong subjective wish to have a TIVAP implanted and signs a consent form (3, 18); and (6) Current guidelines or studies have not reached a consensus on the definition of the “long-term” time frame.

Consistent with the suggestions of Chopra V et al. (19) and Simonov M et al. (20), TIVAP has proven to be a feasible choice for intravenous infusion pathways that need to be utilized for more than 3 months. A study performed by Jiang LT et al. emphasizes the cost-effectiveness of TIVAP when the catheter retention time exceeds 4 months (21). Likewise, numerous clinical guidelines advocate for the adoption of TIVAP for treatment cycles lasting longer than 6 months (16, 22).

### 3.3 Contraindications for the totally implantable venous access port

Contraindications for the TIVAP include (1): Abnormal coagulation function, notably preoperative hypocoagulable or hypercoagulable states, is correlated with a heightened risk of complications after surgery (23). Research has indicated that a prior history of deep vein thrombosis (DVT), thrombosis developing at the puncture site, the presence of tumors, coagulation-related genetic factors, and other high-risk factors conspicuously increase the probability of catheter-related thrombosis post-operatively (24, 25) (2); Uncontrolled bacteremia or local infection at the puncture site (3); Known allergy to infusion port materials (4); Surgery-related areas that have received radiation therapy or local tissue scars that affect the stability of the port; and (5) Comorbid serious chronic diseases that cannot tolerate the procedure.

Contraindications to TIVAP are relative and should be considered in conjunction with the urgency and substitutability of vessel opening.

## 4 Surgical points for setting up the totally implantable venous access port

### 4.1 Venous puncture vessel selection

The anterior chest region typically employs the internal jugular vein, subclavian vein, and the third segment of the axillary vein as the preferred blood vessels for TIVAP procedures. Regardless of the particular vessel selected, it is crucial to abide by a catheter/vein diameter ratio not exceeding 45%, thus minimizing the risk of catheter-related thrombosis (26).

Despite the relatively simple nature of puncturing the internal jugular vein, this procedure has disadvantages, including long subcutaneous tracts, privacy issues, and discomfort caused by neck movement traction. Moreover, the catheter passing across the clavicle and the subsequent 180-degree turn at the venous puncture site increase the risk of thrombosis and local inflammation (27). On the contrary, the subclavian vein approach, although technically more demanding and associated with a higher rate of hemopneumothorax, is also prone to catheter occlusion or rupture due to the pinch-off syndrome (28), thereby restricting its clinical application (29, 30). Since Westcott’s pioneering achievement of axillary vein puncture in 1972, there has been an increasing agreement among experts and a growing body of research supporting ultrasound-guided axillary vein access for TIVAP implantation (31).

The debate regarding the comparative advantages of these three venous access routes remains unresolved, with inconsistent results among studies. A review of the combined data obtained from numerous research showed that the internal jugular vein route has a lower risk of infection (10%) and thrombosis (7%) compared to the subclavian vein route, which is associated with a 21% infection rate and a 22% thrombosis rate. Additionally, the internal jugular vein route showed a significantly lower rate of long-term complications (22%) than the subclavian vein route (49%). These findings align with the conclusions of a related study, which supports the higher probability of both short- and long-term complications in the subclavian vein access group, thus favoring the internal jugular vein route as a preferred option.

However, a study carried out by Liling Han et al. revealed that in a pediatric patient population, the subclavian vein approach had a lower occurrence of catheter occlusion and higher patient satisfaction, and therefore recommended it as the preferred choice for TIVAP implantation in children. Furthermore, a report from Guo et al. suggests that the axillary venous approach is better for reducing complications and postoperative abnormal port retrieval rates compared to the internal jugular and subclavian venous approaches, making it a more favorable choice for clinical application. Chen Tianyou et al. revealed that the left axillary vein approach is less affected by neck and shoulder movements, utilizing a wider angle between the axillary and subclavian veins, which facilitates the puncture and placement of the catheter. It is recommended to use ultrasound real-time guidance for the left axillary vein approach, as it increases the success rate and reduces complications.

It is important to note the growing popularity of ultrasound-guided supraclavicular brachiocephalic vein punctures for implanting TIVAPs in both children and adults in recent years. Anatomically, the brachiocephalic vein forms from the convergence of the internal jugular and subclavian veins, making it the largest of the central veins available for access (32). The catheter-diameter-to-vein-diameter ratio in the brachiocephalic vein is less than 45%, which serves as a preventative measure against catheter-related thrombosis. Additionally, the puncture site in the supraclavicular fossa enhances patient comfort and lowers the risk of infection (33).

In 2019, Xingwei Sun initially reported on the clinical application of ultrasound-guided TIVAPs via the right brachiocephalic vein in adult patients with cancer (34). In the aftermath, Xingwei Sun and his research team launched a detailed series of thorough inquiries into the practical clinical usage of TIVAPs, with a specific emphasis on the brachiocephalic approach, all conducted under the exacting direction of ultrasound technology. Studies led by Xingwei Sun have shown that the brachiocephalic vein method, guided by ultrasound, is as safe and reliable as the internal jugular vein approach (35). Further research by Sun and his team suggests that ultrasound guidance during brachiocephalic vein procedures improves intraoperative navigation and reduces the likelihood of complications during and after surgery (36). Wei Ding and his colleagues have explored the use of this technique in pediatric patients, finding it to be a viable, safe, and effective option (37).

### 4.2 Catheter implantation technology

In accordance with the promotion of the minimally invasive concept, the percutaneous puncture method has gradually become more preferred over the vascular incision technique, which has significantly reduced the occurrence of TIVAP-associated implantation trauma (38). Our previous research used the Seldinger method to insert guide wires enclosed in split sheaths into blood vessels, whereas, in other studies, the cannula-retention method was chosen to minimize the potential harm caused by the insertion of metal guide wires and vascular dilators (39).

### 4.3 Catheter tip positioning technology

The positioning of the catheter tip in intravenous infusion devices is crucial for maintaining infusion stability and reducing the possibility of complications. According to existing research, there is a direct connection between the location of the catheter tip and the occurrence of catheter-related thrombosis (CRT) (40). Currently, an initial international consensus has been reached on the recommended position of the catheter tip, especially within the area ranging from the lower one-third of the superior vena cava to the cavo-atrial junction (CAJ). This positioning approach has been associated with a lower risk of catheter thrombosis and an improvement in infusion efficiency (41). Nevertheless, it should be emphasized that there is still no standardized method for accurately determining this position.

Several experts have unanimously agreed on the use of X-ray fluoroscopy technology for accurate localization of the sixth thoracic cone, which serves as a proxy for the inferior one-third of the superior vena cava to the CAJ (42, 43). Additionally, several authorities have agreed to use the anatomical reference of the two vertebral bodies below the carina as a definite marker for the junction of the vena cava and atrium (44). Moreover, specific studies have proposed a standardized formulaic approach, which involves dividing the height (in centimeters) by 10 and then subtracting 3, for the purpose of localization. An alternative method involves calculating the distance from the puncture point to the right sternoclavicular joint, increased by 7 cm, with a marginal variation of approximately 1 cm based on individual body size (22, 36).

Recent research has shown that, in the absence of fluoroscopic conditions, intracardiac electrocardiography can be used as an effective method for determine the position of the catheter tip (44). Shujun Yang et al. used transesophageal echocardiography (TEE) for visualizing the convergence of the superior and inferior vena cava into the right atrium, thereby localizing the catheter tip precisely. Their findings suggest that TEE provides higher accuracy in localization compared to perspective and formulaic methods. However, it is essential to note that the technical requirements for cardiac ultrasound are strict, and TEE can only be performed under anesthesia, thus limiting its application in various clinical situations (45).

## 5 Complications of the totally implantable venous access port

The complication rate for TIVAP procedures lies within the range of 1.8% to 14.4% (50). Many studies have underscored the need for the participation of trained professionals in the procedure to minimize the risk of complications (18, 22, 46).

### 5.1 Non-specific complications

#### 5.1.1 Catheter malfunctions

Catheter malfunctions include catheter dysfunction, catheter lumen occlusion, catheter misalignment and kinking, pinch-off syndrome, and catheter breakage or dislodgement. The incidence rates of catheter malfunctions fall approximately within the range of 0.8% to 5.0% (47).

When mechanical compression is ruled out as a contributing factor, the swift identification of thrombosis becomes of utmost significance in cases of catheter occlusion. The formation of a fibrin sheath, a vascularized fibrous connective tissue that develops when thrombus adheres to the catheter surface, frequently poses a challenge for blood withdrawal but allows for continuous medication infusion. After meticulous consideration and the exclusion of contraindications for thrombolysis, prompt intracatheter thrombolysis is of prime importance for restoring catheter functionality (48).

Pinch-off-syndrome, a medical condition that can be conclusively diagnosed through imaging methods such as chest X-rays or computed tomography scans, arises due to the limited space between the clavicle and the first rib. It occurs when a catheter is inserted into a blood vessel, which leads to prolonged pressure on the clavicle and first rib. This sustained pressure can cause the lumen to narrow, potentially close, or even rupture. Based on available reports, the incidence of pinch-off-syndrome is estimated to lie between 0.8% and 1% (49).

Catheter breakage or dislodgement, a notable complication of TIVAP, typically stems from incorrect catheter connection, catheter aging, and the occurrence of pinch-off syndrome. Furthermore, it can be provoked by increased abdominal pressure resulting from severe coughing, physical exertion, or repetitive vomiting. The overall incidence of this complication is within the range of 0.1% to 2.1% (50).

#### 5.1.2 Catheter-related thrombosis

CRT, a particular type of venous thromboembolism, has been reported in a considerable number of asymptomatic cases, ranging from 30% to 70%, account for 10% of all deep venous thromboses (DVT) in adults and 50–80% of all DVTs among children. Healthcare professionals should remain highly vigilant, as this form of thrombosis can act as a precursor of infection and pulmonary embolism (51). The incidence of CRT lies within the scope of 2% to 26%. As per the accessible literature, ultrasound is the suggested first-choice diagnostic approach for CRT, and venography can be utilized as an alternative when needed. Several factors influence the occurrence of CRT, including common risk elements like type of tumor and placement of the catheter tip.

However, across the board, there is no consensus on the most efficient treatment scheme for CRT. Many clinicians tend to prescribe anticoagulants like heparin or warfarin depending on whether the catheter is retained or removed (52). Nevertheless, certain randomized controlled trials have indicated that the prophylactic use of warfarin or heparin in cancer patients does not lower the incidence of CRT (53).

#### 5.1.3 Local and systemic infections

Infection remains a major concern related to TIVAP. Due to the complexities of clinical differential diagnosis, TIVAPs are usually removed promptly when there is suspicion of infection, which is a major reason for their removal, with the incidence ranging from 5.6% to 8%. In the case of local infections, appropriate measures include meticulous local wound care or the administration of antibiotics. If the pathogen is identified as *Pseudomonas* or atypical *Mycobacterium*, immediate removal of the catheter is considered a feasible option (54).

Systemic infection, also known as catheter-related bloodstream infection (CRBI), is the leading cause for premature discontinuation of TIVAP catheters or the onset of systemic sepsis. As numerous studies have shown, coagulase-negative staphylococci is the main pathogen in CRBI and can be effectively treated with vancomycin. If fever and blood-borne pathogens persist after three days of standardized antibiotic treatment, especially in the presence of *Staphylococcus aureus* infection, the prompt removal of the TIVAP catheter should be carefully evaluated.

Currently, a unified protocol for the management of TIVAP infection has not been established, particularly regarding the decision to retain or remove the TIVAP in cases of infection. Domestic guidelines generally suggest that for patients with mild symptoms, systemic antibiotics should be administered based on drug sensitivity testing, and local “antibiotic lock” therapy can be used to preserve the catheter. However, in cases where anti-infective therapy is ineffective or infections involving *S. aureus* or *Candida albicans* occur, immediate removal of the TIVAP is recommended (55).

#### 5.1.4 Drug extravasation

Drug leakage from TIVAP has been reported, occurring in 3%–6% of patients receiving TIVAP (56). Upon verification of such an occurrence, the use of TIVAP must be promptly halted, and therapeutic measures should be initiated without delay. It is worth noting that reports on drug extravasation in treatment using TIVAP are relatively scarce, and there are no standardized protocols for managing this issue. Nevertheless, we can draw inspiration from the principles for managing the extravasation of drugs in peripheral veins to formulate effective treatment strategies (57).

### 5.2 Specific complications

In the backdrop of technological progress and proficiency, the incidence of specific complications related to the port in TIVAP procedures has significantly decreased during the period of treatment, dropping to approximately 13%, compared to previously reported complication rates in about 45% of patients (58).

#### 5.2.1 Cystic hematoma

Cystic hematoma is a relatively urgent and common complication related to TIVAP procedures, with an incidence rate varying from 0% to 4.5%. This complication emerges during the TIVAP procedure because of the rupture of capillaries in the subcutaneous or subfascial tissues. The occurrence of cystic hematoma is closely linked to the positioning of the port. Studies have indicated that the incidence of hematoma is greater when the port is implanted in the endothoracic fascia as opposed to when it is placed in subcutaneous tissue.

The causes of cystic hematoma are numerous and complex. Recent research suggests that patients receiving heparin anticoagulation before surgery have a five times higher risk of developing post-surgical cystic hematoma than those undergoing warfarin anticoagulation, and a ten times higher risk compared to those not receiving anticoagulant therapy (59). In cases where bleeding can be effectively managed through hematoma evacuation and drainage, the port can be retained. However, if the hematoma continues to enlarge and the risk of infection increases, prompt removal of the port is necessary (60).

#### 5.2.2 Incision dehiscence

Incision dehiscence, which happens in approximately 3% of cases after a successful TIVAP implantation, refers to the reopening of the surgical incision along the suture line. This complication can be generally classified into mechanical and functional types. For patients with minor wound dehiscence, local wound care and skin flap transplantation are suggested treatment options. In situations where there is severe infection along with wound dehiscence, it is essential to promptly remove the TIVAP and provide suitable antibiotic therapy. After wound debridement and successful healing, the possibility of reimplantation can be re-evaluated (18).

#### 5.2.3 Inversion of infusion port

When the infusion port is standardly placed within a subcutaneous pouch, the occurrence of port reversal is typically relatively rare. Nevertheless, when the size of the pouch exceeds the recommended range, the subcutaneous tissue lacks adequate tightness, or if there is premature movement and stretching by the patient after surgery, it can lead to excessive displacement of the port, causing it to flip, twist, or reverse. We can promptly detect and diagnose port flipping through lateral chest X-ray. In the majority of cases, manual reduction methods can successfully treat port flipping. However, if the flipping persists despite attempts at repositioning, surgical intervention might be required to reshape the pouch and re-anchor the port.

## 6 Totally implantable venous access port standardized maintenance and education

As an expensive intravenous infusion device, TIVAP is of the greatest significance in cancer treatment, demanding the execution of standardized management throughout its entire life cycle. Non-invasive puncture needles should be used following the principle of minimizing harm, emphasizing gentle puncturing techniques, restricting excessive needle movement, and mandating weekly needle replacement (61). Given TIVAP’s extended retention period, home care plays a vital role. We strongly suggest that, between treatment intervals, the catheter be maintained at the hospital on a monthly basis, with regular dressing changes to guarantee wound cleanliness, dryness, and the prevention of infections. During the recovery phase, patients must avoid significant limb movement to protect the surgical outcome. Upon the discovery of abnormalities, such as redness, swelling, pain, or oozing at the TIVAP surgical site, patients are urged to promptly return to the hospital for examination.

## 7 Conclusions

As a crucial intravenous infusion tool for cancer treatment in recent years, TIVAP significantly enhances the survival quality and comfort of patients while offering a comfortable and stable infusion channel. Despite the growing popularity of TIVAP in clinical applications both domestically and internationally, there is still a dearth of international standardized guidelines for it. Therefore, there is an urgent need for more multicenter and prospective clinical studies to provide a deeper exploration of the core issues, such as the optimal selection of implanted blood vessels for TIVAP, catheter tip positioning methods, and the management of catheter-related infections. We believe that the development of unified and standardized TIVAP full life cycle practice guidelines would lead to a reduction in complications and enhance the quality of life of patients.
